# BAF (mSWI/SNF) complex regulates mediolateral cortical patterning in the developing forebrain

**DOI:** 10.3389/fcell.2022.1011109

**Published:** 2022-10-03

**Authors:** Huong Nguyen, Godwin Sokpor, Arpan Parichha, Linh Pham, Nidhi Saikhedkar, Yuanbin Xie, Pauline Antonie Ulmke, Joachim Rosenbusch, Mehdi Pirouz, Rüdiger Behr, Anastassia Stoykova, Beate Brand-Saberi, Huu Phuc Nguyen, Jochen F. Staiger, Shubha Tole, Tran Tuoc

**Affiliations:** ^1^ Institute for Neuroanatomy, University Medical Center, Georg-August-University Goettingen, Goettingen, Germany; ^2^ Faculty of Biotechnology, Thai Nguyen University of Sciences, Thai Nguyen, Vietnam; ^3^ Department of Human Genetics, Ruhr University Bochum, Bochum, Germany; ^4^ Department of Anatomy and Molecular Embryology, Ruhr University Bochum, Bochum, Germany; ^5^ Tata Institute of Fundamental Research, Mumbai, India; ^6^ Max Planck Institute for Multidisciplinary Sciences, Goettingen, Germany; ^7^ Harvard Stem Cell Institute, Harvard Medical School, Boston, MA, United States; ^8^ German Primate Center-Leibniz Institute for Primate Research, Goettingen, Germany

**Keywords:** cortical development, patterning, dorsal telencephalon, cortical hem, hippocampus, BAF (mSWI/SNF) complex, Lhx2

## Abstract

Early forebrain patterning entails the correct regional designation of the neuroepithelium, and appropriate specification, generation, and distribution of neural cells during brain development. Specific signaling and transcription factors are known to tightly regulate patterning of the dorsal telencephalon to afford proper structural/functional cortical arealization and morphogenesis. Nevertheless, whether and how changes of the chromatin structure link to the transcriptional program(s) that control cortical patterning remains elusive. Here, we report that the BAF chromatin remodeling complex regulates the spatiotemporal patterning of the mouse dorsal telencephalon. To determine whether and how the BAF complex regulates cortical patterning, we conditionally deleted the BAF complex scaffolding subunits BAF155 and BAF170 in the mouse dorsal telencephalic neuroepithelium. Morphological and cellular changes in the BAF mutant forebrain were examined using immunohistochemistry and *in situ* hybridization. RNA sequencing, Co-immunoprecipitation, and mass spectrometry were used to investigate the molecular basis of BAF complex involvement in forebrain patterning. We found that conditional ablation of BAF complex in the dorsal telencephalon neuroepithelium caused expansion of the cortical hem and medial cortex beyond their developmental boundaries. Consequently, the hippocampal primordium is not specified, the mediolateral cortical patterning is compromised, and the cortical identity is disturbed in the absence of BAF complex. The BAF complex was found to interact with the cortical hem suppressor LHX2. The BAF complex suppresses cortical hem fate to permit proper forebrain patterning. We provide evidence that BAF complex modulates mediolateral cortical patterning possibly by interacting with the transcription factor LHX2 to drive the LHX2-dependent transcriptional program essential for dorsal telencephalon patterning. Our data suggest a putative mechanistic synergy between BAF chromatin remodeling complex and LHX2 in regulating forebrain patterning and ontogeny.

## Introduction

Telencephalic patterning is crucial for the anatomical and functional designation of various aspects of the forebrain during embryogenesis. The dorsal telencephalon (dTel) is broadly organized into the (cerebral) cortex and the dorsal midline. The cortex is sub-divided into the neocortex and archicortex (the hippocampus), whereas the cortical hem and choroid plexus comprise the dorsal midline [reviewed in (Hébert and Fishell 2008)]. The cortical hem and anti-hem, localized at the border between the dTel and ventral telencephalon (vTel), play opposite instructive roles as telencephalic organizers *via* production of morphogens for the early molecular patterning of the brain [Reviewed in (Kiecker and Lumsden 2012)]. The commitment of portions of the telencephalic neuroepithelium to generate distinct structural and functional telencephalic regions, such as the neocortex and hippocampus, takes place early in forebrain development, i.e., E10.5–E12.5 in mouse (Levitt et al., 1997; Gitton et al., 1999; Mallamaci et al., 2000). The graded exposure of dTel progenitors to morphogens orchestrate forebrain patterning through their ability to regulate downstream patterning modulators, including transcription factors, that play instrumental roles in positional specification of telencephalic structures (Sur and Rubenstein 2005; Mallamaci and Stoykova 2006; Rash and Grove 2006; O'Leary and Sahara 2008).

The cortical hem is a telencephalic structure flanked by the hippocampal primordium and the choroid plexus. It is a prominent source of patterning signals, including those that belong to the Wingless/Int (WNT) (Grove et al., 1998) and the Bone morphogenetic protein (BMP) families (Furuta et al., 1997). Thus, the cortical hem performs crucial roles in both cortical and hippocampal patterning due to its morphogen enrichment (Grove et al., 1998; Lee et al., 2000; Mangale et al., 2008; Caronia-Brown et al., 2014). Furthermore, the cortical hem generates reelin-producing Cajal-Retzius cells that migrate tangentially to populate the marginal zone of the cortex and perform important roles in neocortical and hippocampal lamination (Rice and Curran 2001; Meyer et al., 2002; Takiguchi-Hayashi et al., 2004; Bielle et al., 2005; Forster et al., 2006).

Precise molecular regulation and proper cytoarchitecture are prerequisites for the establishment of the various cortical domains during dTel patterning [reviewed in (Subramanian and Tole 2009)]. A few factors, such as FOXG1, EMX1/2, PAX6, LHX2, FGF, LMX1A and GLI3 (Grove et al., 1998; Bishop et al., 2000; Mallamaci et al., 2000; Bulchand et al., 2001; Shimogori et al., 2004; Shinozaki et al., 2004; Kimura et al., 2005; Muzio and Mallamaci 2005; Mangale et al., 2008; Chizhikov et al., 2010; Godbole et al., 2018), and components of signaling pathways (WNT, FGF, BMP) (Forster et al., 2006; Hébert and Fishell 2008) are fundamental in directing cortical patterning. Despite the essential role of these factors for correct forebrain patterning, the interconnecting mechanistic network involved is largely unknown.

The Brg1/Brm-associated factor (BAF) is the mammalian SWI/SNF complex which is a multi-subunit ATP-dependent chromatin remodeler. It is capable of re-structuring chromatin through histone mobilization and/or recruitment of epigenetic cofactors to modify the epigenetic landscape of chromatin (Sokpor et al., 2021b), leading to regulation of gene expression during brain development [reviewed in (Sokpor et al., 2017)]. We previously developed mouse models of inactive BAF complex. This was achieved *via* double conditional knockout (dcKO) of the scaffolding BAF subunits BAF155 and BAF170 in cortical progenitors, which leads to disassembly of other subunits in the BAF complex and their subsequent elimination by the ubiquitin-proteasome machinery (Narayanan et al., 2015; Bachmann et al., 2016; Nguyen et al., 2016; Nguyen et al., 2018). The aberration of the entire BAF complex function in dcKO or partly in single knockouts of BAF155 or BAF170, disturbs several aspects of neural development, including increase in progenitor proliferation and delamination, reduced neural cell differentiation, delayed/stalled neuronal migration, and decrease in oligodendrogenesis (Tuoc et al., 2013; Narayanan et al., 2015; Bachmann et al., 2016; Nguyen et al., 2016; Tuoc et al., 2017; Narayanan et al., 2018; Nguyen et al., 2018; Abbas et al., 2021; Sokpor et al., 2021a).

Interestingly, the BAF complex and the LIM homeodomain transcription factor LHX2 appear to share common developmental functions. Thus, ablation of BAF complex leads to neurodevelopmental phenotypes similar to that of *Lhx2* deletion in several neural tissues, including the eye and olfactory neuroepithelia. Disturbance of *Lhx2* expression causes misregulation of proliferation and differentiation of the retinal neural progenitors, and defective gliogenesis (Lamba et al., 2008; Tétreault et al., 2009; Balasubramanian et al., 2014; de Melo et al., 2016; de Melo et al., 2018), a phenotype partly recapitulated by BAF complex manipulation in the developing human fetal retina (Lamba et al., 2008). Similarly, the olfactory epithelium of *Lhx2* mutant mice have abnormal progenitor differentiation, deprivation of olfactory receptor gene expression, and loss of neuronal identity leading to perturbed development and diversification of olfactory receptor neurons (Hirota and Mombaerts 2004; Kolterud et al., 2004). Likewise, conditional inactivation of the BAF complex in the olfactory epithelium in mice resulted in loss of LHX2-expressing olfactory receptor neurons and overall dysgenesis of the olfactory epithelium (Bachmann et al., 2016). Notably, both BAF complex and LHX2 are known to regulate neurogenesis during forebrain development in a WNT-signaling dependent manner (Hsu et al., 2015; Nguyen et al., 2018). As a result, the respective mutants displayed reduced cortical size due to decreased cortical neurogenesis (Hsu et al., 2015; Nguyen et al., 2018).

In this study, we show that the BAF complex is a determinant of cortical identity and a suppressor of cortical hem enlargement during forebrain development. Interestingly, we found that the BAF complex interacts with LHX2 and is required for normal LHX2 transcriptional activity in the dTel, suggesting their functional interaction to effect correct mediolateral patterning of the developing forebrain. Particularly, loss of BAF complex resulted in an expansion of the cortical hem at the expense of the medial and dorsolateral cortical primordia, leading to distortion of mediolateral cortical patterning. Additionally, we postulate an evolutionary/developmental significance of the differential expression of BAF complex subunits in mouse and the marmoset cortex and hem. These results highlight the neurodevelopmental role of the BAF complex in regulating the anatomical (regional) and functional organization of the mammalian cerebral cortex.

## Materials and methods

### Animals

Floxed *BAF155* (Choi et al., 2012), floxed *BAF170* (Tuoc et al., 2013), *FoxG1-Cre* (Hebert and McConnell 2000), *Emx1-Cre* (Gorski et al., 2002), and *hGFAP-Cre* mice (Zhuo et al., 2001) were maintained in a C57BL6/J background (license numbers 14/1636 and 16/2330).

Marmoset monkey (*Callithrix jacchus*) were obtained from the self-sustaining colony of the German Primate Center (Deutsches Primatenzentrum; DPZ) and housed according to the standard German Primate Center practice for common marmoset monkeys. Embryonic and fetal stages were obtained surgically by hysterotomy, or hysterectomy (license numbers 42502-04-12/0708 and 42502-04-16/2129) as described previously (Aeckerle et al., 2015; Wolff et al., 2019). All surgical work on the monkeys were performed by a veterinarian with several years of experience in handling and operating marmoset monkeys. A detailed description of experiment with marmoset monkey is provided in Supplementary Material. Animals were handled in accordance with the German Animal Protection Law.

### Plasmids and antibodies

A list of plasmids and antibodies with detailed descriptions is provided in Supplementary Material.

### *In utero* electroporation, and *in vivo* (LHX2) and *in vitro* (LHX2/PAX6) transcriptional activity assay

*In vivo* and *in vitro* transcriptional activity assay were performed as previously described (Pinon et al., 2008; Nguyen et al., 2018). Briefly, LHX2 transcriptional activity *in vivo* was monitored by electroporating brains of E12.5 *BAF15*5^fl/fl^;*BAF170* ^fl/fl^ embryos with a Cre plasmid (or empty plasmid as a control) and the reporter constructs *pGL3-5xLhx2BS-luciferase* or *pGL3-luciferase* (Folgueras et al., 2013) together with *pRL-TK* constructs at a 5:1:0.3 ratio. LHX2 transcriptional activity was measured in the E14.5 mouse cortex. For *in vitro* assay, Neuro2A cells at 1×10^5^ per well density were plated into 24-well plates. Cells were transfected with 0.8 μg of shRNA plasmids (*shBAF155*, *shBAF170*) along with 50 ng of *Pax6-luc* (Kammandel et al., 1999) and 10 ng of *pRL-TK*. Two (2) days post-transfection, cells were then collected for Pax6 transcriptional activity measurement. In all cases, firefly luciferase activity was normalized to that of Renilla luciferase.

### CoIP/mass spectrometry, RNA sequencing

Detailed descriptions were provided previously (Narayanan et al., 2015).

### Immunohistochemistry

After blocking with 5% goat or donkey normal serum, coronal sections of brain or whole head were incubated overnight with primary antibody at 4°C, and the signal was detected with a fluorescent secondary antibody (Alexa Fluor; 1:400; Invitrogen).

### *In situ* hybridization

ISH was carried out as described previously (Tuoc et al., 2009).

### 3D reconstruction

3D images of the cortical hem were constructed using Neurolucida software version 11.03. Every third consecutive sections (10 μ each) of BAF mutant and control brains were imaged in rostro-caudal order. Contours were drawn in each section based on the expression of cortical hem-specific markers. The 3D reconstruction was produced from whole-stack contours. The contours were placed into sets for left and right cortical hem as depicted in Supplementary Movie S1. The hem volume estimation was done by using Neurolucida Explorer v. 11.03.

### Cell counts and quantitative analysis of IHC signal intensity

Immunostaining in IHC images was quantified using anatomically matched forebrain sections. Nucleus-marker positive cells within the pallium of confocal images were counted for comparison. In most cases, cell counts from six matched sections were averaged from three biological replicates (control/dcKO pallium). In many cases, the number of lineage marker cells was quantified using total marker-positive cells alone, or by normalizing to the total number of DAPI+ (nucleus-stained) cells using the following equation: Normalized number = marker-positive cell number/DAPI + cell number. Statistical analyses of histological data were performed using unpaired Student’s *t*-test. All bar graphs are plotted as means ± SD. All statistical tests are two-tailed, and *p*-values are considered to be significant for *α* = 0.05.

### Imaging, quantification, and statistical analyses

All images were acquired with standard (Leica DM 6000) and confocal (Leica TCS SP5) fluorescence microscopes. Images were further analyzed with Adobe Photoshop. IHC and ISH signal intensities were quantified by using ImageJ software. Statistical analyses were done using Student’s *t*-test. All graphs are plotted as mean ± SD.

## Results

### Expression of BAF complex in the embryonic mammalian dorsal telencephalon

**FIGURE 1:**
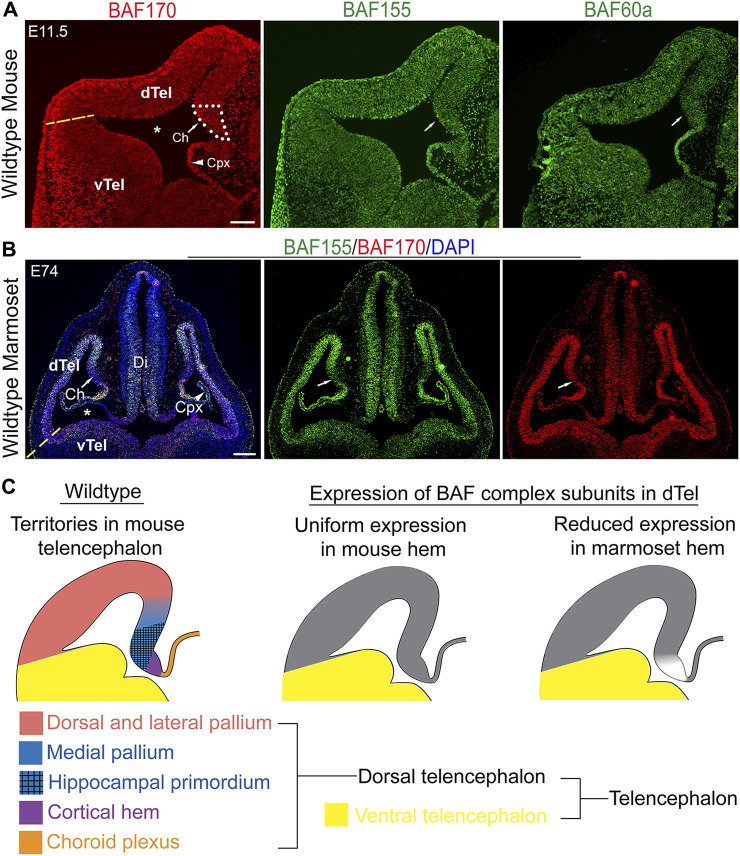
Comparative expression analysis suggests a role for BAF complex in mammalian forebrain patterning. **(A)** Images showing hemi-sections of the E11.5 wild-type mouse telencephalon immunostained for the BAF complex subunits BAF155, BAF170, and BAF60a. An approximation of the border between the dorsal telencephalon and ventral telencephalon is indicated with a yellow dashed line. Medial telencephalic territories, i.e., the cortical hem and choroid plexus, are shown. **(B)** Images of the marmoset whole head showing immunostaining of the E74 telencephalon with BAF155, BAF170 antibodies. Arrow points to the cortical hem area. The lateral ventricle is indicated by an asterisk. Yellow dashed line indicates an approximation of the dorsal and ventral telencephalon boundary. **(C)** Schematics showing territorial designation of the mouse dorsal telencephalon (right panel), and the generalized expression of BAF complex subunits in the mouse (middle panel) and marmoset (left panel) dorsal telencephalon. Abbreviations: dorsal telencephalon (dTel), ventral telencephalon (vTel), diencephalon (Di), choroid plexus (Cpx). DAPI counterstaining is shown. Scale bars: 200 µm in **(A)**, 250 µm in **(B)**.

The expression of BAF complex in the early developing mouse dTel was analyzed by immunohistochemical (IHC) staining of the scaffolding BAF complex subunits BAF155 and BAF170, and an additional BAF complex subunit BAF60a, which together are indicative of BAF complex presence (Narayanan et al., 2015). The examined BAF subunits were observed to be expressed throughout the E11.5 (Figure 1A) and E13.5 control mouse dTel (Supplementary Figure S1A) without an obvious or discernable gradient pattern of expression. The consistency of BAF complex expression is maintained laterally and continues into the vTel. Likewise, the dorsal telencephalic midline structures, cortical hem and choroid plexus, display uniform BAF complex expression (Figure 1A).

On the other hand, comparative analysis of BAF complex expression in a non-human (primate-like) brain model unveiled demonstrable changes in the expression pattern of BAF complex compared with that of mouse. Notably, we observed intense expression of BAF155 and BAF 170 in the E74 marmoset dTel, which becomes reduced or diffused ventrally (i.e., in the vTel) (Figure 1B). Interestingly, there is a discernable abrupt discontinuation or reduction of BAF complex expression in the region of the cortical hem and its resurgence in the adjoining choroid plexus in the marmoset E74 dTel (Figures 1B,C). The observed lack of BAF complex in the marmoset cortical hem contrasts that seen in the developing mouse cortical hem (Figure 1A vs. 1B; Figure 1C). This observation drew our attention to investigating a possible role for BAF complex in driving cortical hem-mediated dTel patterning or morphogenesis during mammalian brain development.

### Loss of BAF155 and BAF170 leads to expansion of the cortical hem

**FIGURE 2:**
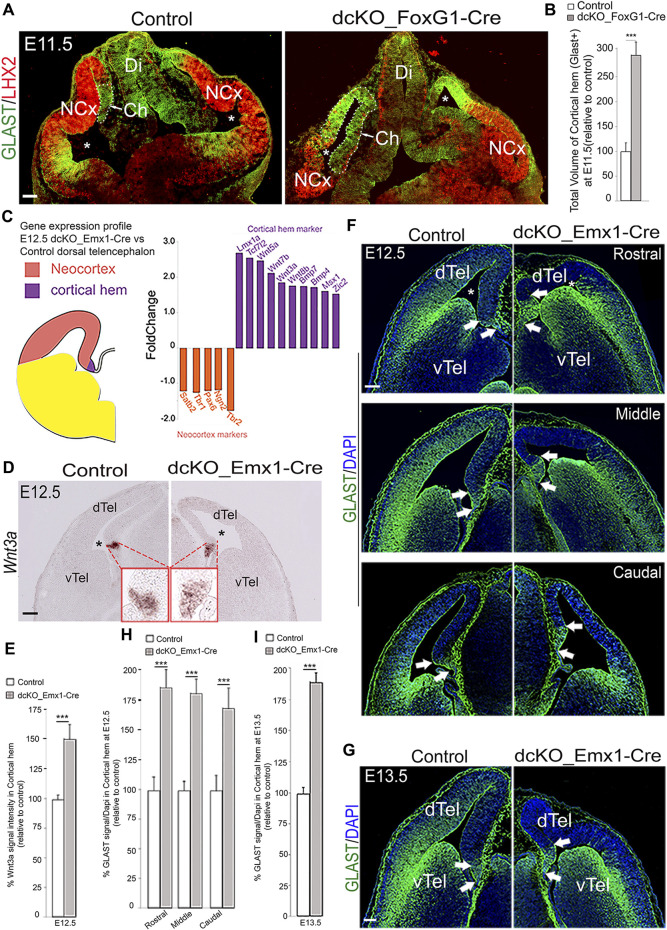
BAF complex ablation causes abnormal cortical hem enlargement. **(A)** Images of the E11.5 mouse whole head sections showing the developing forebrain stained with LHX2 and GLAST antibodies to reveal the presumptive neocortex and the cortical hem (delineated with stippled lines) of the control and *dcKO_Foxg1-Cre* telencephalon, respectively. **(B)** Bar chart indicating the statistical difference between the estimated total volume of control and *dcKO_Foxg1-Cre* cortical hems. **(C)** Schematic showing demarcation of the neocortex from the cortical hem in hemisection of the mouse telencephalon. Results of RNA sequencing of the E12.5 control and *dcKO_Emx1-Cre* cortical tissues indicating fold change of downregulated neocortical marker genes (orange bars) and upregulated hem maker genes (purple bars) in the *dcKO_Emx1-Cre* are graphically represented. **(D)** Micrographs showing *in situ* hybridization in the E12.5 control and *dcKO_Emx1-Cre* dTel with the RNA probe and hem marker *Wnt3a*. **(E)** Bar graphs indicating the statistical differences between the estimated size of the control and mutant hem using Wnt3a signal intensity measurement. **(F,G)** Images showing rostral to caudal sections of the E12.5 **(F)** and mid-section of the E13.5 **(G)** control and *dcKO_Emx1-Cre* brains immmunostained with the GLAST antibody. White arrows point to the boundaries of the cortical hem revealed by intense GLAST staining. **(H,I)** Bar graphs indicating the statistical differences between the estimated size of the control and mutant hem in the respective micrographs with GLAST staining at E12.5 (rostral–caudal) **(F)** and E13.5 **(G)**. Where shown, sections are counterstained with DAPI (blue). The lateral ventricle is indicated by an asterisk. Arrows point to the cortical hem. Results are presented as mean ± SD. Unpaired Student’s *t*-test: ***p < 0.0005; *n* = 4–6; * denotes level of significance; Scale bars = 100 µm in **(A,D,G)**, 50 µm in **(F)**. Abbreviations: NCx, neocortex; Ch, cortical hem; Di, Diencephalon; dTel, dorsal telencephalon; vTel, ventral telencephalon.

We examined the telencephalic phenotype of the *dcKO_Foxg1-Cre* mutant brain in which the BAF complex is conditionally abolished in the mouse telencephalon by deleting both BAF155 and BAF170 subunits (Bachmann et al., 2016; Nguyen et al., 2016) with the Foxg1-Cre driver being active from E8.5 on (Hebert and McConnell 2000). At stage E11.5, the GLAST-immuno-delimited cortical hem in the mutant forebrain was markedly expanded beyond its spatial confinement as compared with control (Figures 2A,B; white arrowed, dotted lines). We performed quantitative analysis of the 3D-reconstructed GLAST-stained hems and found that the E11.5 *dcKO_Foxg1-Cre* cortical hem had undergone about 3-fold volumetric enlargement (Figure 2B; Supplementary Movie S1). Thus, the *dcKO_Foxg1-Cre* cortical hem extended ectopically beyond its normal tiny location at the end of the medial pallial wall into the neuroepithelial domain of the presumptive cortex (Figure 2A). By applying immunostaining against the cortex selective marker, LHX2, it was evident that the size of LHX2-marked cortical primordium was reduced in the *dcKO_Foxg1-Cre* dorsal pallium as compared with control (Figure 2A).

To elucidate BAF complex function during formation of the dTel and specifically in the context of cortical hem-mediated dTel patterning, we crossed mice harboring floxed alleles of *BAF155* (Choi et al., 2012) and *BAF170* (Tuoc et al., 2013) (i.e., *BAF155* ^*fl/fl*^ and *BAF170* ^*fl/fl*^, respectively) with the dTel neuroepithelium-specific *Emx1-Cre* to generate *dcKO_Emx1-Cre* (Narayanan et al., 2015), in which Cre activity is detectable in the presumptive cortex from E10.5 onwards (Gorski et al., 2002). Thus, unlike in the *dcKO_Foxg1-Cre* telencephalon (Figure 2A), in which BAF complex is broadly deleted at the initial stage (E8.5) of telencephalon formation (Xuan et al., 1995; Martynoga et al., 2005; Bachmann et al., 2016), the *Emx1-Cre* practically restricted the loss of BAF complex to the dTel at the onset of neurogenesis (Gorski et al., 2002). Indeed, IHC analysis with antibodies against BAF155 and BAF170 revealed overt loss of BAF complex in the *dcKO_Emx1-Cre* dTel by E11.5 (Supplementary Figure S1).

We proceeded with characterizing the dTel phenotype of the *dcKO_Emx1-Cre* forebrain by initially reexamining data from our previously performed gene expression profiling of the *dcKO_Emx1-Cre* dTel containing both neocortex and cortical hem at embryonic stage E12.5 (Figure 2C) [RNA-seq data sheet in (Narayanan et al., 2015); GSE106711]. This analysis revealed that 1,723 transcripts were downregulated and 102 upregulated in the E12.5 *dcKO_Emx1-Cre* dTel. Notably, many genes important for cortical identity (e.g., *Pax6, Ngn1, Ngn2, Tbr1 and Satb2*) were downregulated, whereas expression levels of several known cortical hem-specific genes including *Wnt3a*, *Wnt5a* and *Bmp* were upregulated (Figure 2C). We validated our RNA-seq data by examining the expression pattern and level of *Wnt3a*, which is confined to or concentrated in the cortical hem at early stages of cortical development (Figure 2D; [Lee et al., 2000; Louvi et al., 2007)]. From our ISH analysis, we found that the *Wnt3a* expression level and area of expression were increased in the mutant dTel as compared with control at E12.5 (Figures 2D,E), thus corroborating the upregulated expression of *Wnt3a* seen in our RNA-seq data (Figure 2C).

Besides measuring the expression pattern of upregulated genes, we also examined expression of the radial glial markers GLAST and BLBP, which can delimit the cortical hem (Figures 2F,G; Supplementary Figure S2). In early cortical development, between E11.5–E13.5, GLAST expression displays a lateral^high^ to medial^low^ gradient in the neocortex, but with localized high expression level in the cortical hem (Figures 2F,G, demarcated with arrows). Analysis of the 3D-reconstructed E12.5 and E13.5 *dcKO_Emx1-Cre* cortical hem, as done for *dcKO_Foxg1-Cre* hem analysis (Figure 2B; Supplementary Movie S1), revealed nearly 2-fold expansion by volume as compared with control (Figures 2H,I). Such expanded *dcKO_Emx1-Cre* hem was observed at all rostrocaudal levels of the cortical hem system (Figures 2F,H). Thus, our histological analyses suggest that the increased expression of hem marker genes revealed in the RNA-seq data is likely due to cortical hem enlargement in the *dcKO_Emx1-Cre* forebrain at E12.5. That notwithstanding, the expanded cortical hem and associated dysmorphic cortical phenotypes are less severe in *dcKO_Emx1-Cre* in comparison to the *dcKO_Foxg1-Cre* dTel (Figures 2F,G vs. Figures 1A,B). This is consistent with the report of Godbole et al. (2018), in which deletion of *Lhx2* at progressively later stages resulted in a corresponding decrease in expansion of the hem.

Of note, *Emx1_Cre*-mediated single knockout of either *BAF155* (*BAF155 cKO*) or *BAF170* (*BAF170 cKO*) did not elicit an overt expansion of cortical hem (Supplementary Figure S3) as seen in the *dcKO_Emx1-Cre* mutants (Figures 2D–H). This implies that the function of *BAF155* and *BAF170* is necessary to orchestrate normal development (suppression) of the cortical hem system. The requirement of BAF complex in temporal regulation of cortical hem size during corticogenesis was suggested by the outcome of the experiment in which BAF complex was ablated later in cortical development as in *dcKO_hGFAP-Cre* mutants in which Cre is late-active, namely around E13.5 (Zhuo et al., 2001; Nguyen et al., 2018). Notably, we observed no significant change in the size of the E15.5 cortical hem (intensely stained with GLAST antibody, and without PAX6 labeling) in the *dcKO_hGFAP-Cre* dTel as compared with control (Supplementary Figures S3C–E; unpaired *t*-test, *p*-value = 0.926; *n* = 4). It implies that the modulatory function of the BAF complex in hem morphogenesis is exerted within a limited early developmental time window as demonstrated in the *FoxG1-Cre* and *Emx1-Cre* mutants (Figure 2). The temporal regulation of hem size was also reported in previous studies in which hem size was unaffected after later deletion of factors known to regulate hem size at early cortical development stages (Muzio and Mallamaci 2005; Hanashima et al., 2007; Mangale et al., 2008; Godbole et al., 2018).

Taken together, our RNA-seq results and elaborate histological analyses indicate that the loss of BAF complex in the dTel neuroepithelium at the onset of cortical neurogenesis leads to aberrant expansion of the cortical hem. This suggests that the BAF complex may act as a suppressor of hem fate during early forebrain development.

### Medial pallium expands and encroaches laterally in the absence of BAF complex

**FIGURE 3:**
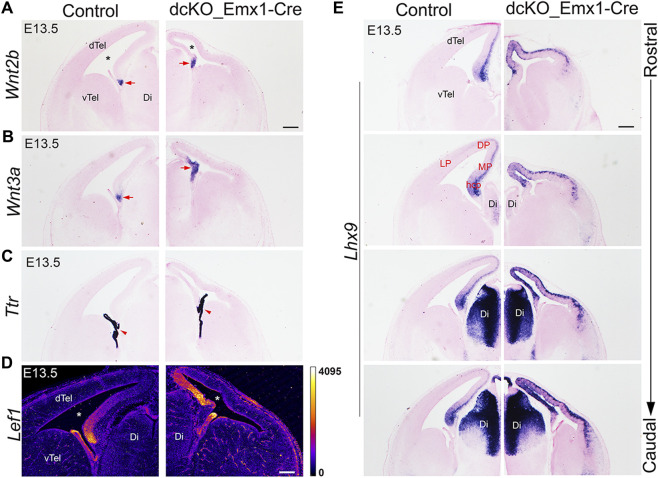
Early loss of BAF complex in the developing dorsal telencephalon distorts cortical patterning. **(A–E)** Images showing *in situ* hybridization or immuostaining in the control and *dcKO_Emx1-Cre* E13.5 mouse (dorsal) telencephalon with the riboprobes *Wnt2b* **(A)** and *Wnt3a* **(B)** to mark the cortical hem, *Ttr* (transthyretin) to mark the choroid plexus **(C)**, LEF1 antibody to stain the medial cortex **(D)**, and *Lhx9* for revealing the cortical hem and medial cortex in rostral to caudal sections **(E)**. The lateral ventricle is indicated by an asterisk. Arrow points to cortical hem. Arrowhead points to choroid plexus Abbreviations: dTel, dorsal telencephalon; vTel, ventral telencephalon Ch, cortical hem; Di, Diencephalon. LP, lateral cortex; DP, dorsal cortex; MP, medial cortex; hcp, hippocampal primordium. Scale bar: 200 µm.

Since loss of BAF complex in the dTel neuroepithelium resulted in abnormal expansion of the cortical hem, we turned our attention to possible impact on mediolateral patterning in the developing BAF mutant telencephalon. We applied ISH analysis to examine the expression pattern of medial dTel (archicortical) markers on serial coronal sections of the E13.5 control and *dcKO_Emx1-Cre* forebrain (Figure 3). Upon close examination, we observed abnormal patterning of the dorsal midline structures and neocortex in the *dcKO_Emx1-Cre* mouse dTel. Per *Wnt2b* and *Wnt3a* ISH, we noticed mild expansion of the *dcKO_Emx1-Cre* cortical hem as compared with control (Figures 3A,B). However, the choroid plexus, which is the ventral continuum and derivative of the cortical hem (Louvi et al., 2007), did not present with any noticeable morphological alteration in the *dcKO_Emx1-Cre* when compared to control, as indicated by ISH staining with the choroid plexus-specific marker transthyretin (*Ttr*) (Figure 3C; [Herbert et al., 1986; Grove et al., 1998; Monuki et al., 2001)].

Furthermore, immunostaining for LEF1 and ISH with the RNA probe *Lhx9*, both of which reveal medial cortex/pallium identity, indicated drastic lateral expansion/extension of the medial cortex at the expense of the dorsolateral cortex in the *dcKO_Emx1-Cre* dTel (Figures 3D,E). Normally, by E12.5, LHX9 is strongly expressed in the hippocampal primordium and the expression intensity tapers from medial to lateral cortex. Around E13.5, LHX9 is expressed in the ventricular zone of the hippocampal primordium [Figure 3E; (Bulchand et al., 2003)] and in pioneer postmitotic neurons (mainly preplate cells) (Bertuzzi et al., 1999) of the wild-type medial cortex/pallium. However, *Lhx9* expression is observed to fill the whole of the medial cortex and is further strongly expressed in the lateral cortex of the *dcKO_Emx1-Cre* dTel (Figure 3E). This deviation in *Lhx9* expression from the aforementioned normal pattern supports the idea of abnormal patterning of the *dcKO_Emx1-Cre* dTel. It means that the expanded cortical hem and lateral extension of the medial cortex resulted in predominance of the medial pallium in the entire dTel with more severity in the caudal region (Figure 3E; Supplementary Figure S4).

Put together, the data presented here point to a putative function of the BAF chromatin remodeling complex in directing cortical patterning during mouse forebrain development.

### BAF complex and hippocampal specification

Existing evidence demonstrates that ectopic hippocampal fields can be induced by an ectopic cortical hem (Mangale et al., 2008), indicating that the hem is an organizing center sufficient in the specification and development of the hippocampal primordium. We previously reported that BAF155 and BAF170 are required for the development of the mouse hippocampus (Nguyen et al., 2018). These findings prompted us to investigate the implication(s) of the expanded cortical hem, as observed in this study, for the specification and/or morphogenesis of the hippocampal anlagen.

**FIGURE 4:**
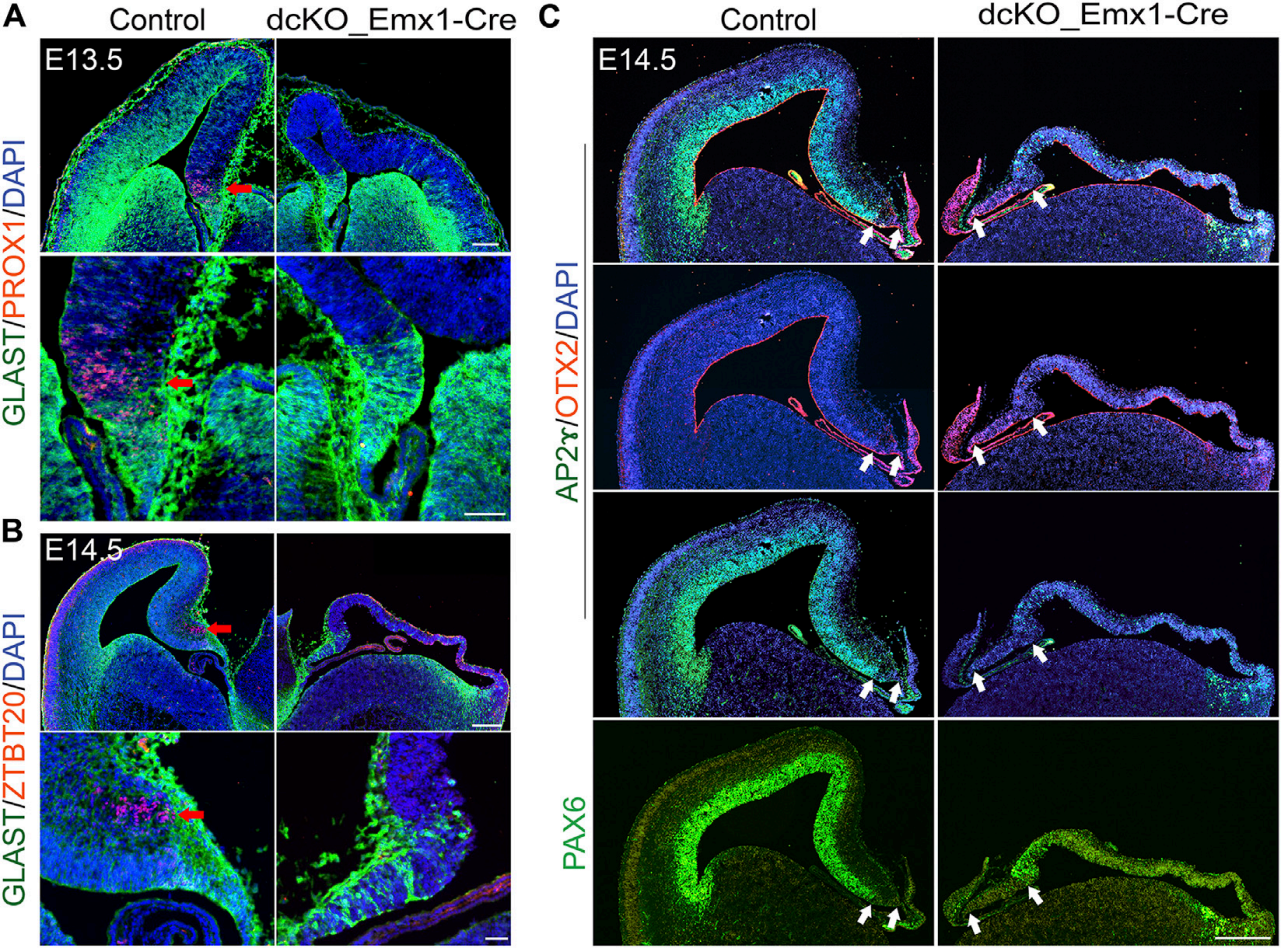
Loss of BAF in dTel perturbs hippocampal formation and neocortical patterning. **(A)** Immunohistochemical micrographs showing hemi-section of the control and *dcKO_Emx1-Cre* mouse brain at E13.5 stained with GLAST and PROX1 antibodies. Panels of close-up images highlight the presence and absence of the PROX1+ dentate gyrus cells in the control and BAF complex mutant medial pallium, respectively. Red arrow points to PROX1 staining. **(B)** Micrographs showing hemi-section of the E14.5 mouse brain stained with GLAST and ZBTB20 antibodies in control and *dcKO_Emx1-Cre*. Panels of close-up images highlight the presence and absence of the ZBTB20 + hippocampal neurons in the control and BAF complex mutant hippocampal primordium, respectively. Red arrow points to ZBTB20 staining. **(C)** Images showing profound loss of cortical mass and patterning associated with the expanded hem in the E14.5 *dcKO_Emx1-Cre* dTel as compared with control. The applied antibodies AP2ɣ and PAX6 stain the cortical neuroepithelium/ventricular zone to reveal the unstained cortical hem, while the hem is stained by OTX2. White arrows indicate the size of the cortical hem. Sections are counterstained with DAPI (blue). Scale bars = 250 µm [in upper panel of **(A)**], 50 µm [in lower panel of **(A)**], 500 µm [in upper panel of **(B)**], 50 µm [in lower panel of **(B)**], 500 µm **(C)**.

Consistent with our findings that deletion of BAF complex components caused progenitor proliferation (Nguyen et al., 2018), the dcKO_Emx1-Cre dTel at E13.5/E14.5 is obviously thinner than controls, with only a few postmitotic neurons. Nonetheless, we examined whether these neurons have been specified to acquire a hippocampal identity. Immunostaining for dentate gyrus marker PROX1 or hippocampal marker ZBTB20 (Xie et al., 2010; Iwano et al., 2012; Rosenthal et al., 2012; Nielsen et al., 2014; Nguyen et al., 2018) revealed no detectable labeling in the vicinity of the cortical hem identified by GLAST staining (Figures 4A,B). Yet, HuCD- and TBR1-expressing postmitotic neurons are present in the E13.5 medial pallium (data not shown). The observed lack of ZBTB20 staining in the BAF complex mutant cortex (Figure 4B) is counterintuitive because ZBTB20 expression is upregulated in the absence of BAF complex (Wang et al., 2021).

To further assess the functional integrity of the expanded *dcKO_Emx1-Cre* cortical hem, we performed immunostaining for REELIN, which can be used to identify Cajal-Retzius cells. These cells arederivatives of the cortical hem (Rice and Curran 2001; Takiguchi-Hayashi et al., 2004; Bielle et al., 2005; Liu et al., 2018) andcontribute to cortical arealization and laminar patterning *via* several secreted factors, including REELIN (Frotscher et al., 2009; Causeret et al., 2021). It was observed that the BAF mutant cortical marginal zone was less populated with reelin-expressing cells compared with control, which may partly be due to elevated cell death as indicated by REELIN staining colocalization with cleaved Caspase 3, a marker for apoptosis, in the mutant marginal zone (Supplementary Figure S5). Of note, such REELIN-expressing cells were seen to have accumulated in the region of the prospective hippocampal anlage and in the cortical hem of the *dcKO_Emx1-Cre* mutant (Supplementary Figure S5), which is indicative of their impaired migration.

Using PAX6 and AP2ɣ (Pinto et al., 2009) as indicators of cortical identity, we observed that loss of BAF complex in dTel engendered massive cortical shrinkage and cortical integrity perturbation by mid-embryonic corticogenesis (E14.5) in the *dcKO_Emx1-Cre* cortex as compared with control (Figure 4C). The severely dysmorphic BAF mutant cortex is seen to have lost the medial to lateral neuroepithelial patterning revealed by lack of PAX6 or AP2ɣ expression in the ventricular zone, which is normally enriched in the wild-type (control) mouse cortical neuroepithelium (Figure 4C). Meanwhile, the cortical hem, marked by OTX2 and without PAX6 or AP2ɣ staining, remained expanded in the E14.5 *dcKO_Emx1-Cre* dTel as compared with control (Figure 4C).

Importantly, the non-specification or aplasia of the hippocampus and abnormal alteration of the cortical structure in *dcKO_Emx1-Cre* forebrain partially phenocopies the *Lhx2* null dTel (Bulchand et al., 2001; Chou et al., 2009; Godbole et al., 2018). Taken together, these findings indicate that the BAF complex is necessary for preserving cortical identity and modulates correct formation of the cortical hem needed for hippocampal specification and/or morphogenesis.

### BAF155 and BAF170 interact with the cortical hem suppressor LHX2 during early cortical development

**FIGURE 5:**
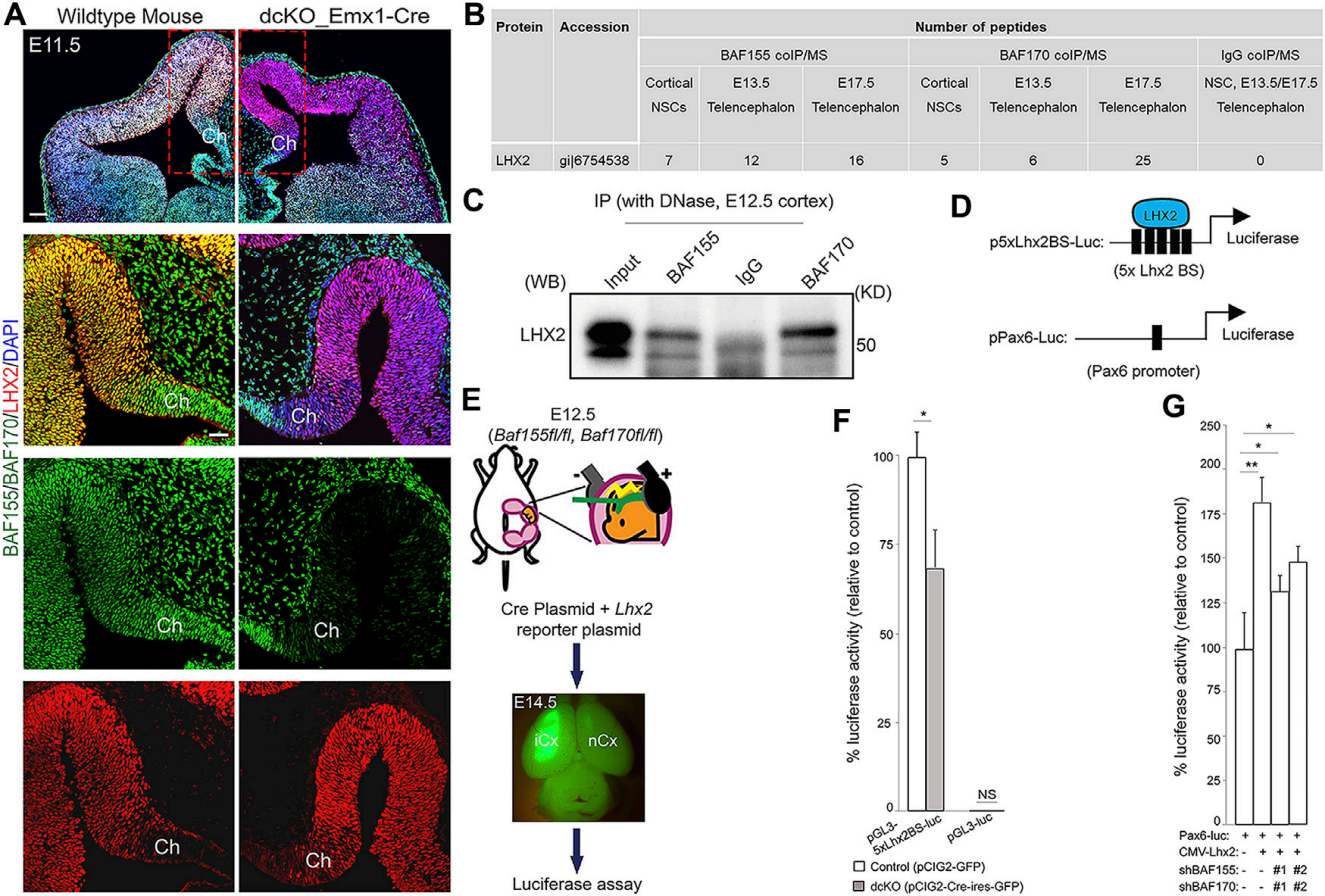
BAF complex interacts with LHX2 in the neocortical neuroepithelium. **(A)** Immunomicrographs showing the E11.5 control and BAF complex mutant mouse telencephalon stained for BAF155, BAF170, and LHX2. Colocalization of BAF complex is indicated by yellow signal. A close-up view of the medial pallium and cortical hem is shown. **(B)** Image of table indicating the number of LHX2 peptides purified from immunoprecipitates of BAF155 and BAF170 obtained from NS5 cell protein extracts, and from the E13.5 or E17.5 dorsal telencephalon protein extract. IgG coIP/MS was used as control. **(C)** Interaction of BAF155 and BAF170 with LHX2 was confirmed by coIP/western blot analyses of E12.5 dorsal telencephalic tissue. **(D)** Illustrations showing the plasmids (*p-5xLhx2BS-luciferase* and *pPax6-Luc*) used for the luciferase assay. The multiple (5x) *Lhx2* binding sites (BS) for augmenting LHX2-dependent promoter activity is depicted in *p-5xLhx2BS-luciferase*, whereas *pPax6-Luc* has one *Lhx2* binding sites. **(E)** Image showing scheme for *in vivo* luciferase assay. **(F,G)** Bar charts representing the statistical comparisons of **(F)** *In vivo* LHX2-mediated luciferase activity in the control or *dcKO_Emx1-Cre* cortex, and **(G)** *In vitro* PAX6-mediated luciferase activity in culture Neuro2A cells in the presence or absence of BAF155, BAF170 or LHX2 permutationally as indicated. In both cases, two variants of *shRNAs* (#1 and #2) were used to knockdown BAF155 and BAF170. Unpaired Student’s *t*-test: *p < 0.05, **p < 0.005; NS stands for not significant; * denotes level of significance; *n* = 6; Results are presented as mean ± SD. Abbreviations: Ch, cortical hem; iCx, injected cortex; nCx, non-injected cortex.

The similarities in the hem expansion upon loss of either LHX2 (Bulchand et al., 2001) or BAF155/170 (this study) motivated us to examine whether these factors participate in a common regulatory pathway or interact with each other. We found extensive co-expression (colocalization) of BAF155 and BAF170 with LHX2 in the E11.5 control presumptive cortical neuroepithelium (Figure 5A). However, lack of BAF complex in the early developing dTel did not cause obvious downregulation of LHX2 expression in the cortical neuroepithelium (Figure 5A; Supplementary Figures S2A,C). This indicates that the BAF complex does not, at least in the examined stages of dTel development (E11.5 and E13.5), have a major influence on the expression level of LHX2 in the presumptive cortex.

We explored the molecular interaction partners of the BAF complex that control formation of the cortical hem and modulate hem-dependent establishment of other dTel structures. To this end, we performed co-immmunoprecipitation (coIP) in cell lysates obtained from the E13.5 or E17.5 telencephalic tissue from wild-type mice, and cultured NS-5 neural stem cells (NSC) using antibodies against BAF155 and BAF170 followed by mass spectrometry (MS) (Narayanan et al., 2015; Nguyen et al., 2018). Many factors essential for neural morphogenesis were identified as protein interaction partners of both BAF155 and BAF170 subunits of the BAF complex (data not shown). Strikingly, we found that LHX2, a determinant of cortical identity and suppressor of hem fate (Bulchand et al., 2001; Monuki et al., 2001; Mangale et al., 2008; Roy et al., 2014), was bound to BAF155 and BAF170 in cultured NSCs, and in the early (E13.5) and late (E17.5) developing forebrain as compared with control (IgG coIP/MS) (Figures 5B,C; *p* score = 1.00).

To examine eventual functional interaction between BAF complex and LHX2, and its neurodevelopmental relevance in the context of hem morphogenesis and cortical formation, we investigated whether the interaction between BAF155/BAF170 and LHX2 influences LHX2-dependent transcriptional activity in the developing mouse cortex. For this purpose, we used an *Lhx2*-dependent reporter vector (*pGL3-5xLhx2BS-luciferase*), containing five *Lhx2* (5x *Lhx2*) binding sites (BS) upstream of a luciferase reporter and an empty vector without *Lhx2* binding site (*pEV-luciferase*) as a negative control (Figure 5D) (Folgueras et al., 2013). To eliminate the function of BAF155 and BAF170, the reporter plus Cre-expressing plasmids were *in utero* co-electroporated into the E12.5 cortex of *BAF155* ^*fl/fl*^ *; BAF170* ^*fl/fl*^ embryos (Figure 5E). We then examined isolated tissue samples from the cortex using a luciferase assay. Our analysis indicated that BAF complex knockout in the dTel or pallium significantly diminished the reporter activity of *pGL3-5xLhx2BS*-luciferase, but not that of the control plasmid lacking the *Lhx2* binding motif (Figures 5E,F). This suggests that BAF155 and BAF170 are together required for activation of LHX2-dependent transcriptional activity.

Alternatively, it is possible that BAF complex might cooperate with LHX2 to regulate the expression of genes governing establishment of cortical identity or patterning. In cortical progenitors, LHX2 is reported to directly regulate PAX6 expression by binding to its promoter (Hou et al., 2013; Shetty et al., 2013; Hsu et al., 2015). Given that PAX6 is a key regulator of cortical progenitor identity and patterning (Bishop et al., 2000; Stoykova et al., 2000; Toresson et al., 2000; Yun et al., 2001; Pinon et al., 2008), we reasoned that its activity may be under the influence of a BAF complex-LHX2 regulatory axis during cortical formation. To verify such a possibility, we first confirmed that *Pax6* is downregulated in the dcKO dTel using IHC analyses (Supplementary Figures S2A,C). We then performed luciferase reporter assays to determine how the expression of LHX2 and BAF complex regulate the activity of a *Pax6* promoter vector, which contains an *Lhx2* binding site (Figure 5D; Kammandel et al., 1999; Hou et al., 2013). The *Pax6* promoter vector driving luciferase expression (*Pax6-Luc*) was transiently transfected into Neuro2A cells together with a combination of *Lhx2*-expression (*CMV-Lhx2*) and *BAF155/BAF170*-silencing vectors (*shBAF155* and *shBAF170*; Figure 5G). After lysate collection and luminescence quantification, the results from the reporter assay revealed that, as compared with control (in *Pax6-Luc + CMV-EV* condition), LHX2 (in *Pax6-Luc + CMV-Lhx2* condition) significantly regulates promoter activity of *Pax6* (Figure 5G). However, upon *BAF155/BAF170* dual knockdown using short-hairpin RNA (*shBAFs*) constructs, the level of luciferase activity reduced (*Pax6-Luc + CMV-Lhx2 + shBAFs* condition), implying that both LHX2 and BAF155/BAF170 are required for maintaining the promoter activity of *Pax6* (Figure 5G).

**FIGURE 6:**
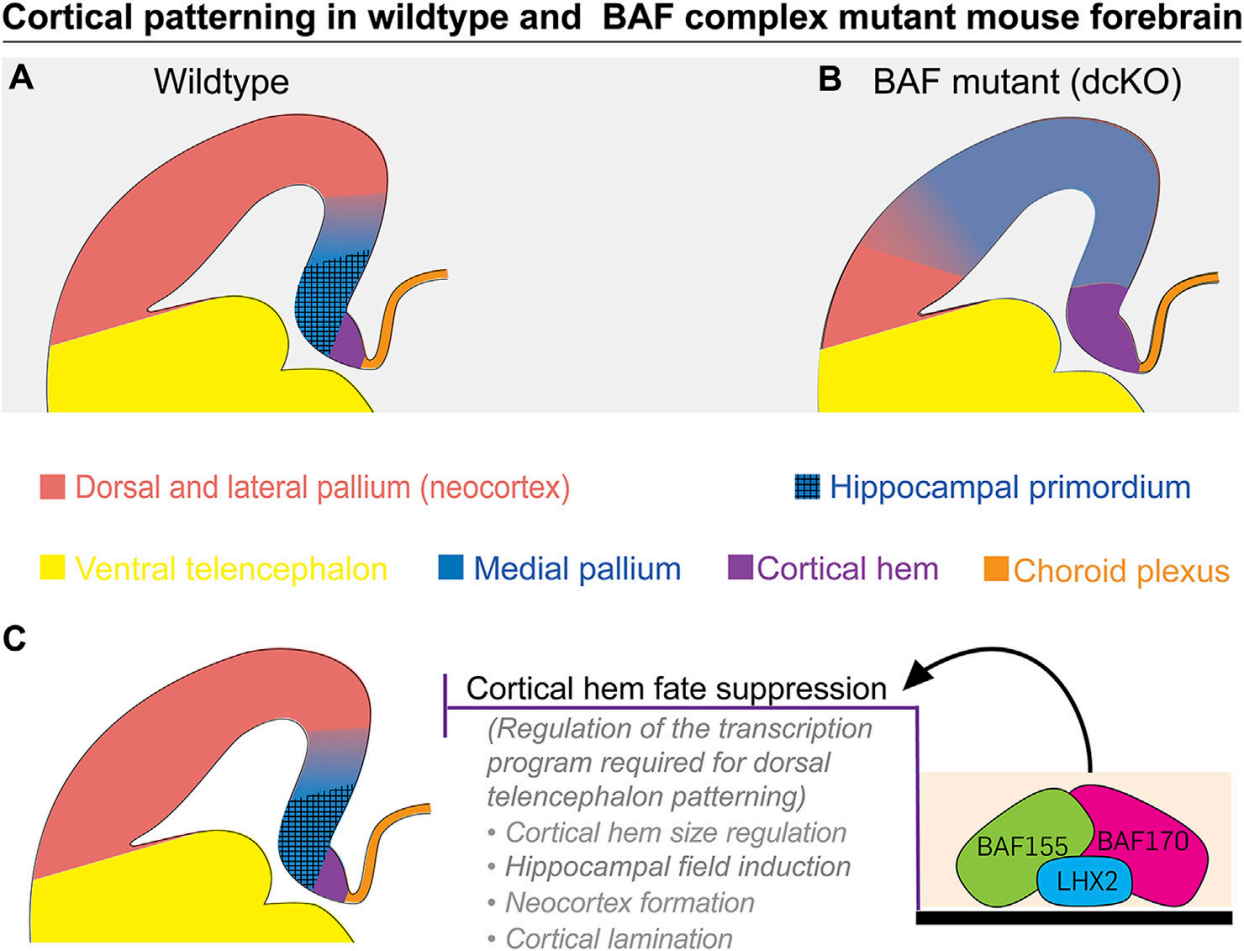
Schema depicting how dorsal telencephalon patterning is perturbed by BAF complex mutagenesis. **(A)** Illustration shows normal regional designation of the dorsal telencephalon (in wild type). The cortical hem, hippocampus, medial cortex/pallium, and neocortex are properly specified and placed in the wild-type dorsal telencephalon. **(B)** Cartoon depicting disturbance of cortical patterning due to lack of BAF complex in the dorsal telencephalic neuroepithelium (in *dcKO_Emx1-Cre*). The hem and medial cortex are seen expanded at the expense of the hippocampus and neocortex, making the *dcKO_Emx1-Cre* dorsal telencephalon have predominance of medial cortex identity. The choroid plexus is not observably affected by BAF complex ablation. **(C)** A putative mechanism involving the interaction between the scaffolding BAF complex subunits BAF155 and BAF170, and the transcription factor LHX2 is put forward as a previously unknown regulatory system for driving proper cortical hem formation and patterning of the dorsal telencephalon.

Altogether, the results of our protein-protein interaction analyses and luciferase assay suggest that the telencephalon patterning regulatory factor LHX2 plausibly acts in concert with BAF complex to orchestrate forebrain development. This finding affords description of a possible molecular basis of the involvement of BAF complex in regulating cortical hem fate and/or morphogenesis *via* functional cooperation with LHX2 and, by extension, its downstream effectors of dorsal telencephalic patterning (Figure 6).

## Discussion

During development, the dTel is organized into structural domains that correlate with specific functional areas. The cortical hem and anti-hem are embryonic organizers located at the medial and lateral edge of the dTel, respectively. The cortical hem is a hub for morphogen signaling known to regulate areal patterning of the embryonic cortical neuroepithelium [reviewed in (Subramanian et al., 2009)]. Absence or abnormal morphology of the cortical hem or molecular manipulations of related morphogenetic factors therein have been linked to various telencephalic perturbations of the developing brain, including cortical mis-patterning (Xuan et al., 1995; Lee et al., 2000; Martynoga et al., 2005; Yoshida et al., 2006; Mangale et al., 2008; Chizhikov et al., 2010; Caronia-Brown et al., 2014; Godbole et al., 2018). However, our understanding of correlating activity of chromatin regulators to the functionality of specific morphogens that drive forebrain patterning is far from clear.

In this study, we focused on determining the role of the ATP-dependent chromatin remodeler BAF complex in regulating forebrain patterning with emphasis on the forebrain hem. Previous studies identified the BAF (mSWI/SNF) complex to play pivotal roles in many aspects of cortical development, including neural progenitor specification, proliferation and differentiation, and maturation of postmitotic neural cells [Reviewed in (Sokpor et al., 2017)]. To expand our understanding of the molecular regulators that ensure the establishment and maintenance of cortical structures and functions, we spatiotemporally inactivated the multimeric chromatin remodeling BAF complex *via* deletion of its scaffolding subunits BAF155 and BAF170 by using *Foxg1-Cre*, *Emx1-Cre* and *hGFAP-Cre*. By this means, we demonstrate in this current study that the BAF complex function is instructive for patterning of the mammalian dTel (Figure 6).

### BAF complex suppresses expansion of cortical hem

We observed that *in vivo* loss of BAF complex during early stages of cortical formation (i.e., under *Foxg1-* and *Emx1-Cre* drivers) caused abnormal expansion of the cortical hem and medial cortex to the detriment of the hippocampal complex and mediolateral cortical identity. The LHX2+ presumptive cortex, although specified in the BAF complex mutant brain, appeared dysmorphic and lateralized. Cortical patterning was seen to be progressively lost from rostral to caudal levels of the telencephalon due to exaggeration of the medial cortical identity or lopsided medial cortical specification. This outcome points to possible distortion of the blueprint for cortical neuroepithelial patterning due to BAF complex ablation and highlights potential abolishment of the secondary organizer function of the expanded cortical hem. Such secondary patterning activity by hem may be necessary for modulating neocortical arealization to yield the various functional cortical areas.

Given the important role of the cortical hem in dTel patterning, we think the BAF complex likely regulates the extent of the cortical hem to modulate cortical patterning. This notion is consolidated by the observation that although the hem is expanded in the absence of optimal BAF complex function, the size of the choroid plexus, which is derived from the hem (Louvi et al., 2007) is not affected. The demonstrable effect of BAF complex disruption on the cortical hem argues its selective regulatory role therein, and/or in the adjoining neocortex. It is also conceivable that the expanded hem is improperly configured, leading to its incompetence in orchestrating arealization of the adjacent cortical primordium or in executing other hem functions.

Our findings convey the idea that the BAF complex likely suppresses inherent expansion tendencies of dTel midline structures, excluding the choroid plexus, during cortical patterning. However, the said suppressive capacity of the BAF complex is likely exerted at early cortical development stages, perhaps until E12.5, as loss of BAF complex from E13.5 achieved in *dcKO_hGFAP-Cre* mutants did not lead to an observable hem or medial cortex expansion, albeit hippocampal formation was perturbed in such mutants (Nguyen et al., 2018). The effect of BAF complex deletion on hem expansion is consistent with outcome of other studies in which genetic manipulation of factors such as *Foxg1*, *Lhx2*, *Lhx2/Pax6* (double deletion), and *Nf2* in early development caused abnormal expansion of the mouse dTel midline structures and associated cortical patterning deviations (Bulchand et al., 2001; Monuki et al., 2001; Vyas et al., 2003; Muzio and Mallamaci 2005; Mangale et al., 2008; Lavado et al., 2013; Godbole et al., 2017). It would be interesting to find out the impact on cortical patterning following focal silencing of the BAF complex in the nascent hem using *Lmx1a-Cre* driver (Chizhikov et al., 2010; Fregoso et al., 2019).

The discernable graded expression of BAF complex in the telencephalon and its distinctive dramatic downregulation in the cortical hem of marmoset, which are not obvious in the mouse cortex, supports a more complex role for BAF complex in driving primate brain morphogenesis. The observations may be adaptive modification or refinement of BAF complex expression that subserves additional functions in the evolved marmoset dTel and hem. In a broader context, these observations may have evolutionary implication for primate cortical patterning and development, especially that the BAF complex has been identified to interact with and augment the transcriptional activity of the well-known cortical patterning regulator LHX2 (Bulchand et al., 2001; Monuki et al., 2001; Mangale et al., 2008).

### Implication of BAF complex and LHX2 interaction for suppression of cortical hem expansion

The formation of cortical hem is regulated by the activity of several factors, including the transcription factor LHX2 and FOXG1 (Godbole et al., 2018). Previous studies reported cortical hem expansion with concomitant massive loss of the cortical identity due to complete or constitutive ablation of *Lhx2* (Bulchand et al., 2001; Mangale et al., 2008). We show here that LHX2 is an interaction partner of the BAF complex. Furthermore, we found that cortical phenotype caused by silencing of BAF complex (this study) resembles that of *Lhx2*−/− mutant (Mangale et al., 2008; Roy et al., 2014), suggesting that both factors are co-dependent in directing telencephalic patterning. We infer from our luciferase experiment that the identified interaction may be a potentiating kind to suppress hem expansion and preserve cortical identity. In further support of such a scenario, we found that the BAF complex promotes the LHX2 target gene *Pax6*, which has a pivotal role in cortical patterning (Bishop et al., 2000; Stoykova et al., 2000; Kimura et al., 2005; Godbole et al., 2017). Moreover, either factor (BAF complex or LHX2) alone is unable to afford sufficient suppression to prevent abnormal expansion of the cortical hem in the absence of the other, hence signifying possible functional cooperation between the BAF complex and LHX2. Together, our new findings disclose the possibility that a correlation between transcription factor activation with specific epigenetic chromatin remodeling machinery act in concert to provide a suppressive function preventing the uncontrolled enlargement of the hem into the normal cortical territory.

The results from the hem phenotype analysis during development in the LHX2- and BAF complex-ablated forebrain, revealed early temporal overlap of their function in effecting hem size restriction. Whereas loss of BAF complex in the dTel under the *Emx1-Cre* driver, with Cre activity beginning from E10.5 (Guo et al., 2000; Gorski et al., 2002), resulted in cortical hem expansion (this study), loss of *Lhx2* at E10.5 or later did not have any impact on hem size compared to control (Godbole et al., 2018). Thus, it appears that the repressive function of the BAF complex on hem fate persists slightly beyond the apparent end point of the LHX2-dependent hem size restriction. In that line of reasoning, we argue that the BAF complex is sufficient in limiting hem expansion and may partly substitute for or complement the function of LHX2 in preventing the uncontrolled expansion of the cortical hem during later or active periods of hem morphogenesis.

Overall, we suggest that the chromatin remodeling BAF complex, partly through interaction with LHX2, engages in the regulatory network that ensures the specification and morphogenesis of the mouse cortical hem, and its placement at the medial edge of the dTel (Figure 6). The pattern of BAF expression in the marmoset telencephalon is suggestive of a similar role in the primate brain. Further investigation is needed to establish the precise role of the BAF complex and its interaction partners necessary for orchestrating development of the highly evolved primate dTel through regulating hem morphogenesis and functionality.

## Data Availability

The datasets presented in this study can be found in online repositories. The names of the repository/repositories and accession number(s) can be found below: https://www.ncbi.nlm.nih.gov/, GSE106711.
