# Supplementary material for: BAF (mSWI/SNF) complex regulates mediolateral cortical patterning in the developing forebrain
Source: Front Cell Dev Biol. 2022 Oct 3;10:1011109. doi: 10.3389/fcell.2022.1011109 (PMC9573979; doi:10.3389/fcell.2022.1011109)
Supplement: Supplementary file 3 [file DataSheet1.docx]

**SUPPLEMENTARY INFORMATION**


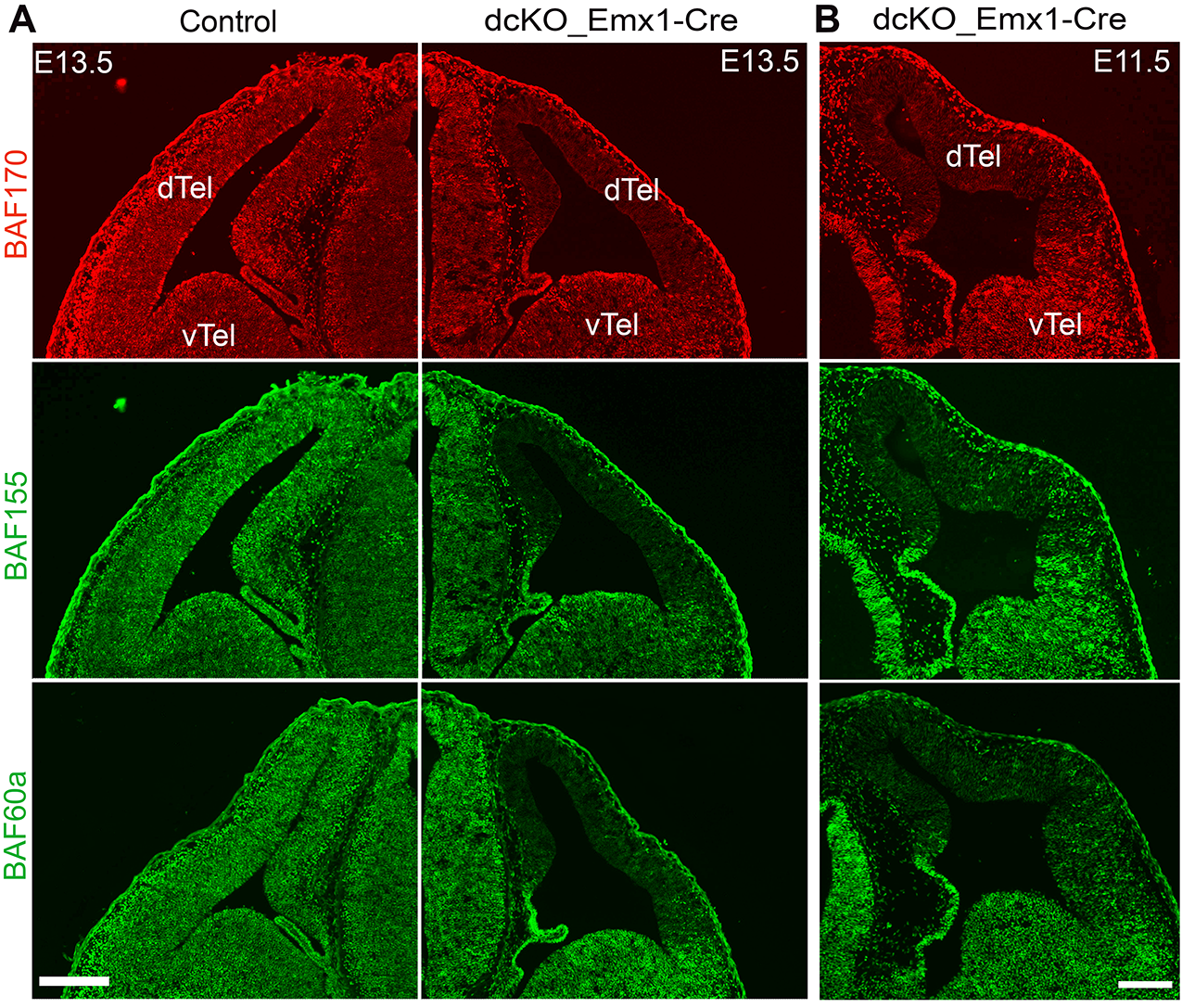


**Figure S1: Emx1-Cre-mediated deletion of BAF155/BAF170 causes early loss of BAF complex in the mouse dorsal telencephalon**

**(A and B)** Images of the E11.5 and E13.5 mouse telencephalon immunostained for the BAF complex. Loss of fluorescence signals of the BAF complex subunits BAF155, BAF170, and BAF60a (to exemplify loss of other BAFs) in the dorsal telencephalon of dcKO_Emx1-Cre compared with control indicate efficiency of the genetic ablation of the BAF complex via Emx1-Cre-mediated double condition knockout of BAF155 and BAF170. Scale bars: 200 µm


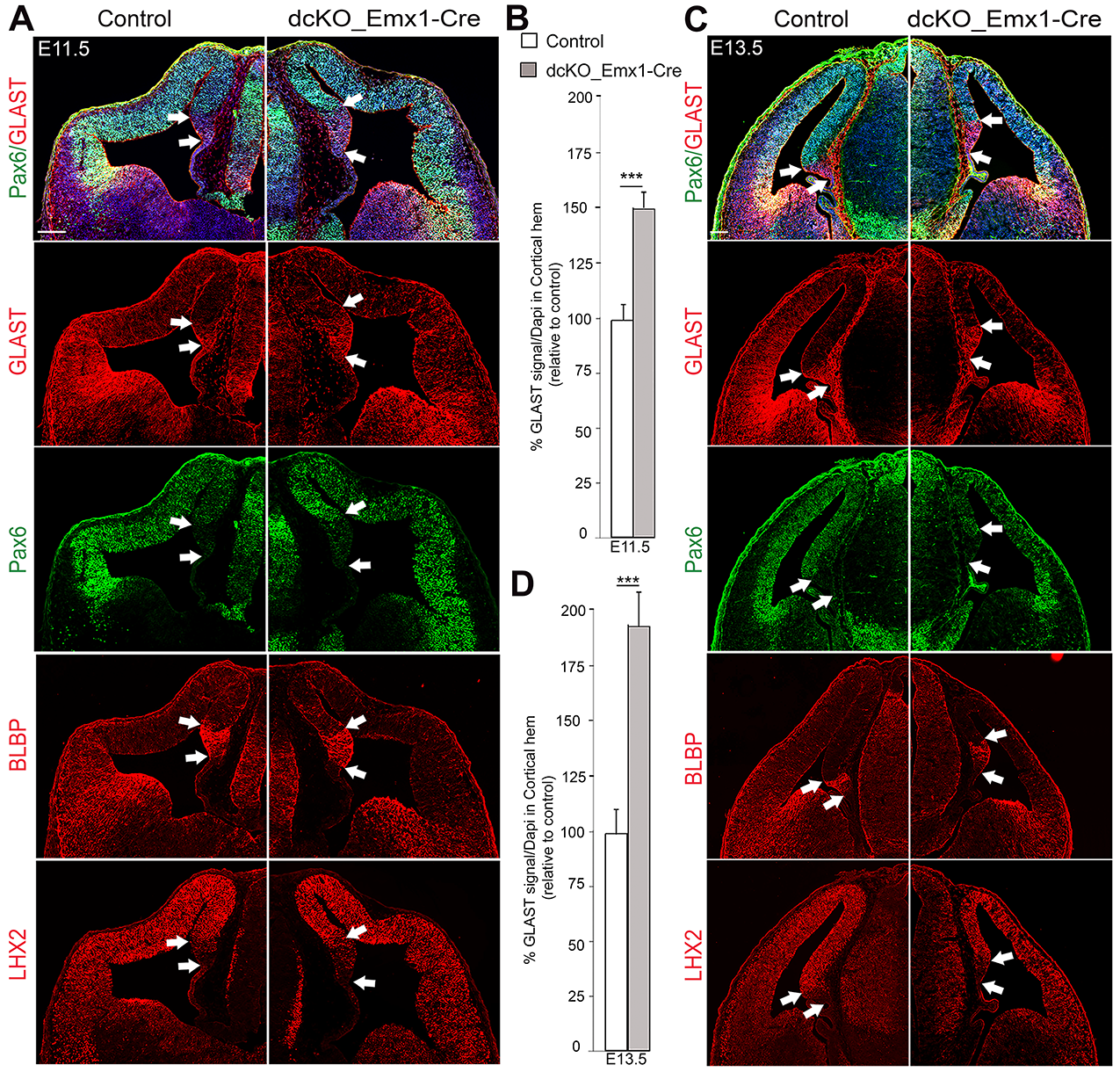


**Figure S2: Expansion of the cortical hem occurs early in corticogenesis following ablation of BAF complex**

**(A and C)** Images showing the E11.5 and E13.5 mouse telencephalon immunostained for GLAST, BLBP, PAX6 and LHX2 to reveal the normal (control) and expanded dcKO_Emx1-Cre cortical hem. Pax6 and LHX2 show the cortical hem identity by their lack of expression therein. The white arrows indicate the full extent of the cortical hem. **(B and D)** Bar charts indicating the statistical differences between the control and dcKO_Emx1-Cre hem size at E11.5 and E13.5. Sections are counterstained with DAPI (blue) where shown. Unpaired Student’s *t*-test: ****p* < 0.0005; * denotes level of significance; n = 4–6; Scale bar: = 100 µm. Results are presented as mean ± SD.


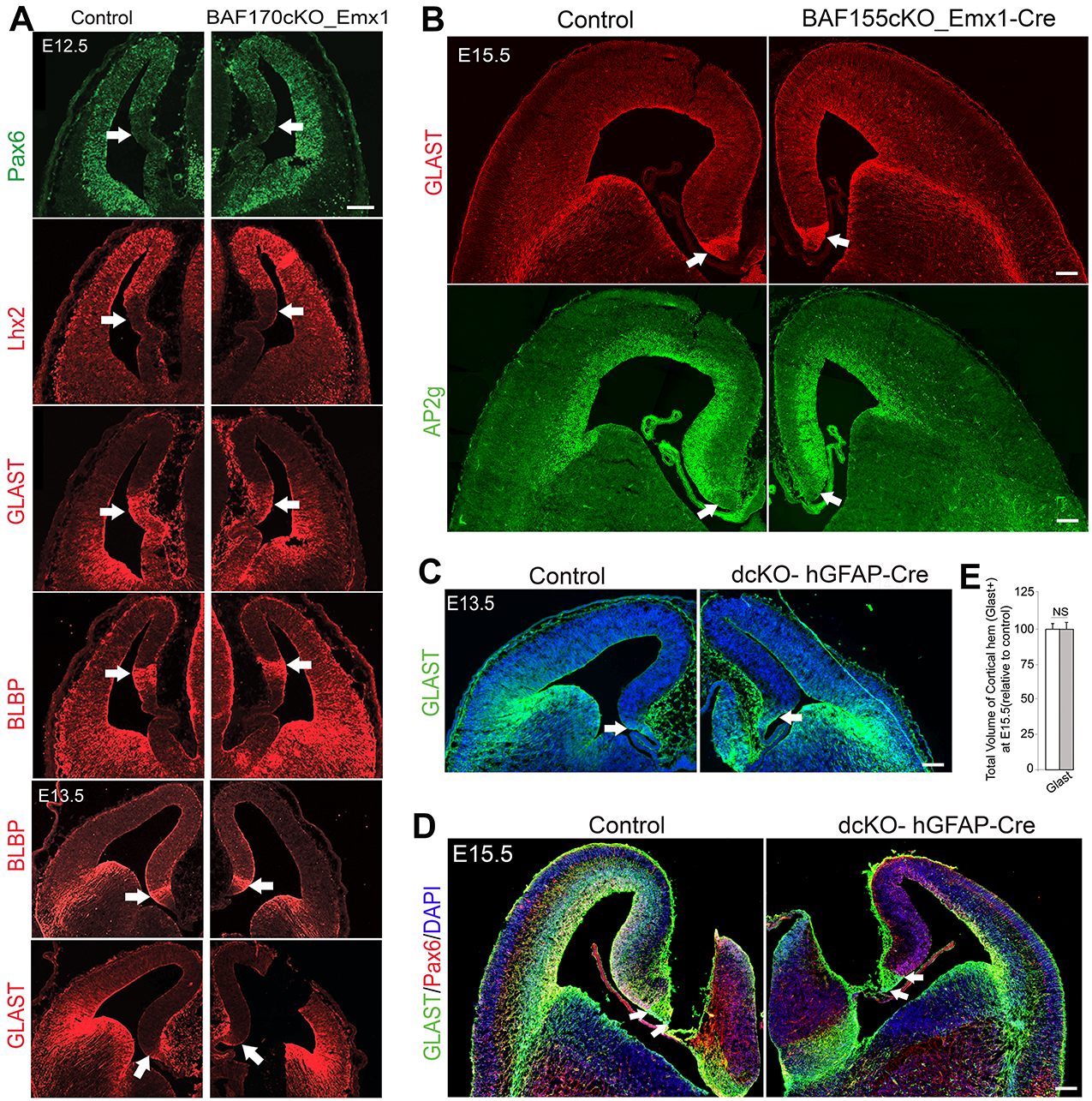


**Figure S3: Single or late double knockout of BAF155 and/or BAF170 does not cause cortical hem expansion**

**(A, B)** Immunomicrographs showing unaltered cortical hem size which is revealed by hem markers following Emx1-Cre-mediated single conditional knockout of BAF170 (**A**) or BAF155 **(B)** in the mouse dorsal telencephalon at the specified developmental stages. **(C, D)** Images showing the normal cortical hem size in both control and the dcKO_hGFAP-Cre dorsal telencephalon at E13.5 (C) and E15.5 (D). (E) Bar chart showing no significant difference in hem size following the late loss of BAF complex in developing cortex. White arrows point to the cortical hem. Sections are counterstained with DAPI (blue). Scale bar = 100 µm (in A), 100 µm (in B and C), 400 µm (in D).


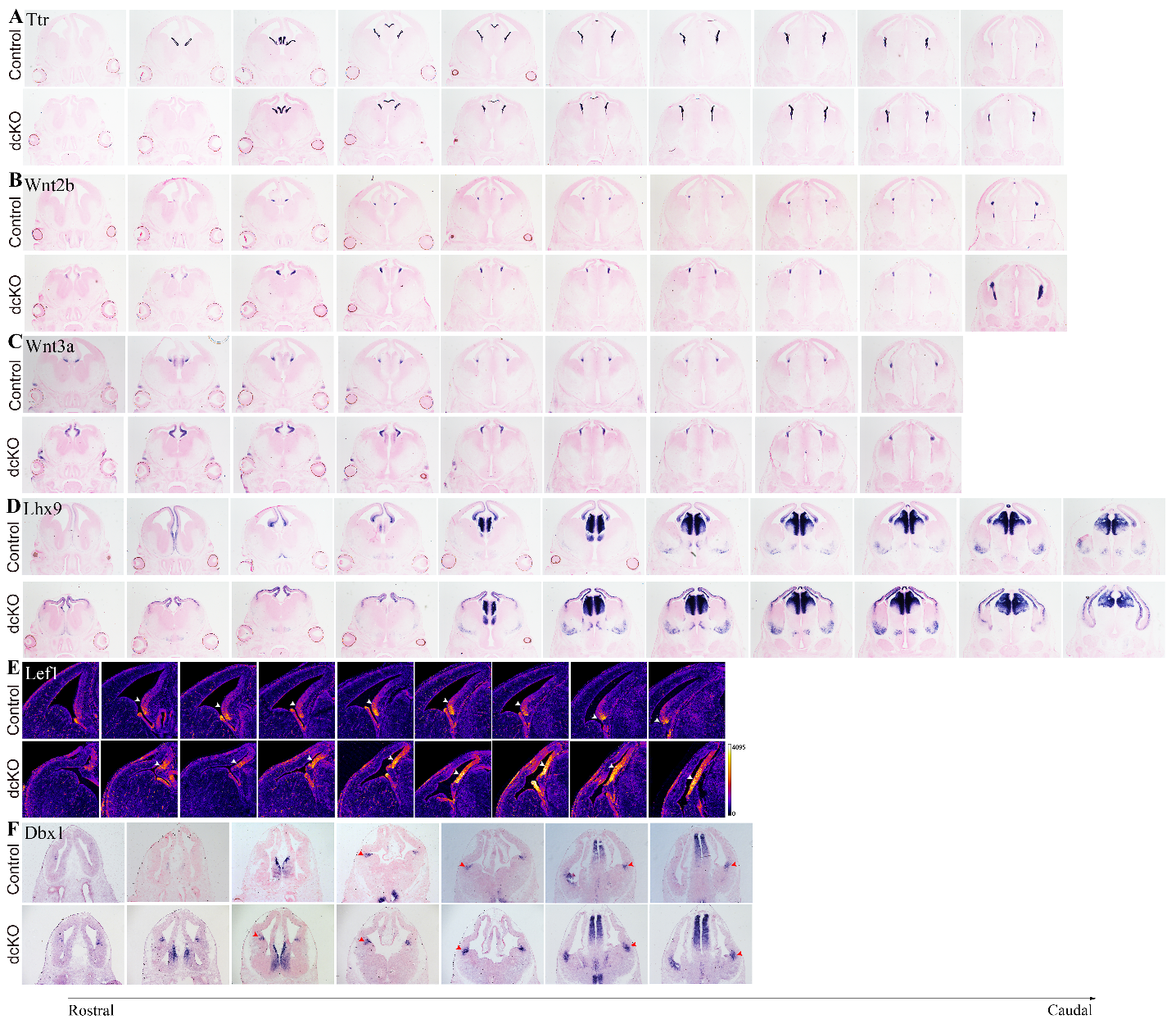


**Figure S4: The dorsal telencephalon is not properly patterned when BAF complex is silenced in the developing mouse cortex**

**(A–F)** Rostrocaudal serial micrographs showing the E13.5 (**A–E**) and E12.5 (**F**) control and dcKO_Emx1-Cre telencephalon RNA-probed or immunostained for *Wnt3a* (**A**)/*Wnt2b* (**B**), *Ttr* (**C**), *Lhx9* (**D**)/LEF1 (**E**), and *Dbx1* (**F**) to label the cortical hem, choroid plexus, parts of the medial cortex, and anti-hem, respectively.


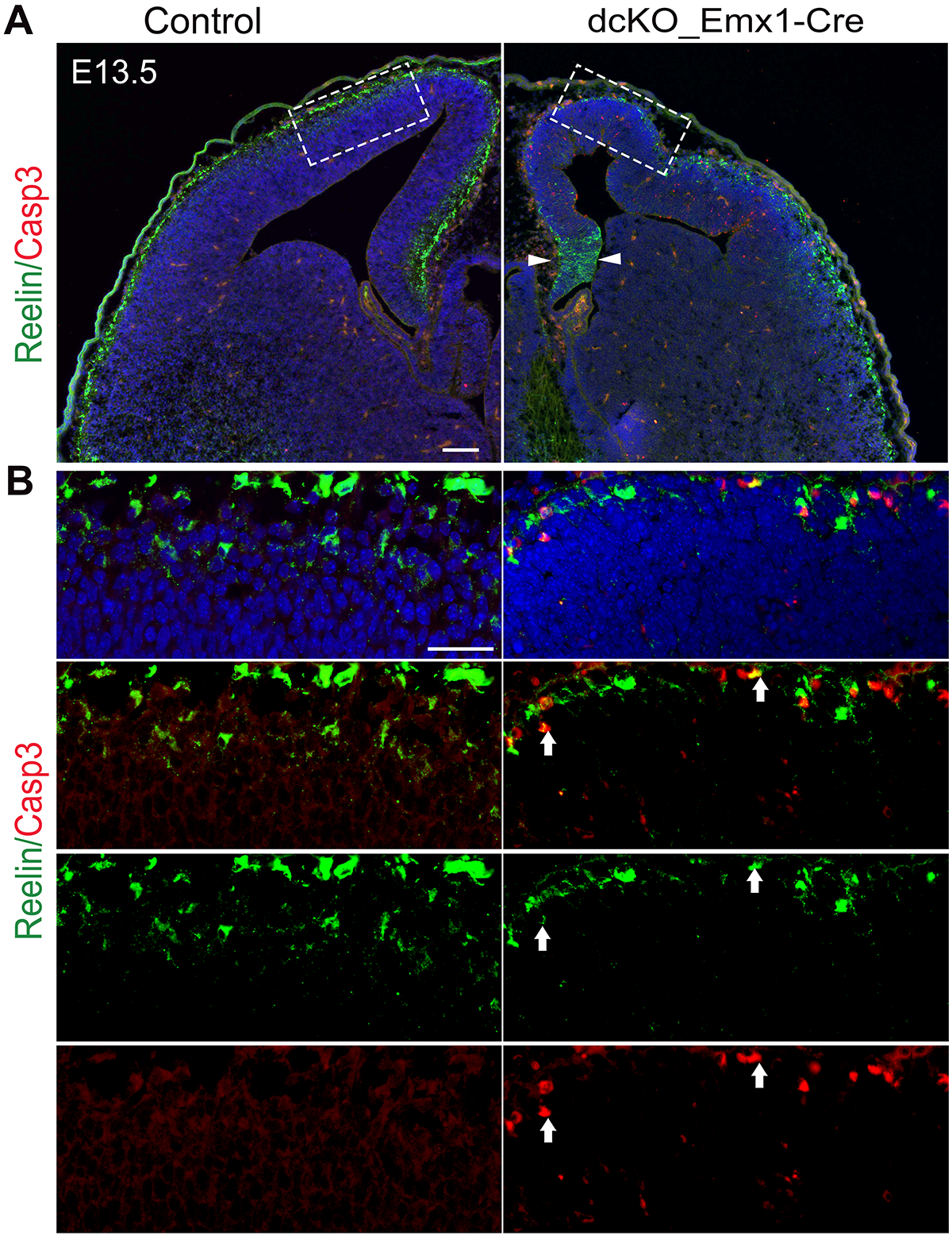


**Figure S5: Distribution and survival of hem-derived reelin-producing cells are impaired due to loss of BAF complex**

**(A, B)** Images of the E13.5 telencephalon immunostained for REELIN to label hem-derived Cajal Retzius cells and CASP3 to mark apoptotic cells. Arrow heads point to accumulated reelin producing cells in the BAF complex mutant cortical hem (A). The frequent colocalization (yellow signal) of reelin and Casp3 staining (white arrows) is indicative of increased apoptosis of the Cajal-Retzius cells produced by the dcKO_Emx1-Cre cortical hem (B). Where shown, sections are counterstained with DAPI (blue). Scale bar = 100 µm (in A), 50 µm (in B).

**Movie S1:** The video shows an example of the reconstructed serially sectioned (E11.5 *dcKO_FoxG1-Cre*) mouse whole head (brain) used for volumetric estimation of the cortical hem colored yellow in the animation and distinguished from the rest of the cortex marked in red. The brain encasement is marked blue.

**Supplemental experimental procedures**

##### ***Plasmids***

Plasmids used in this study: *pCIG2-ires-eGFP*, *pCIG2-Cre-ires-eGFP* (gift from Dr Francois Guillemot, NIMR London); *pGL3-*5x*Lhx2*BS-luciferase is a plasmid containing five *Lhx2* binding sites (BS) upstream of a luciferase (Luc), *CMV-Lhx2* is an expression plasmid contains *Lhx2* cDNA, which was received as a gift from Dr. Elaine Fuchs, the Rockefeller University, New York ([Folgueras et al. 2013](#_ENREF_2)), *Pax6-Luc* (containing *Pax6* promoter upstream of a luciferase (Kammandel et al. 1999). *shBAF155*, *shBAF170* are RNA interfering (silencing) plasmids used to knockdown BAF155 and BAF170 as previously described (Tuoc et al. 2013; Narayanan et al. 2018; Xie et al. 2019; Bi-Lin et al. 2021).

***Antibodies***

The following polyclonal (pAb) and monoclonal (mAb) primary antibodies used in this study were obtained from the indicated commercial sources: AP2γ mouse mAb (1:100; Abcam), BAF170 rabbit pAb (Bethyl), BAF170 rabbit pAb (Sigma), BAF155 rabbit pAb (1:20; Santa Cruz), BAF155 mouse mAb (1:100; Santa Cruz), BLBP rabbit pAb (1:200; Chemicon), CASP3 rabbit pAb (1:100; Cell Signaling), GLAST pig pAb (1:500; Frontier), CidU rat pAb (1:100; Accurate), Ki67 rabbit pAb (1:50; Vector), SOX2 mouse mAb (1:100; R&D Systems), PROX1 rabbit pAb (1:1000; Covance), PAX6 mouse mAb (1:100; Developmental Studies Hybridoma Bank), PAX6 rabbit pAb (1:200; Covance), TBR1 rabbit pAb (1:200; Abcam), ZBTB20 rabbit pAb (1:50; Sigma), and RFP rabbit pAb (1:10000; Biomol/Rockland), BAF60a, Reelin, LHX2 ([Folgueras et al. 2013](#_ENREF_2)) (gift from Dr. Elaine Fuchs, the Rockefeller University, New York).

Secondary antibodies used were horseradish peroxidase (HRP)-conjugated goat anti-rabbit IgG (1:10000; Covance), HRP-conjugated goat anti-mouse IgG (1:5000; Covance), HRP-conjugated goat anti-rat IgG (1:10000; Covance), and Alexa 488-, Alexa 568-, Alexa 594- and Alexa 647-conjugated IgG (various species, 1:400; Molecular Probes).

***Generation of dcKO mutants***

To conditionally eliminate *BAF155* and *BAF170* in the entire telencephalon, early cortical progenitors, or late cortical progenitors, we used the telencephalon-specific *FoxG1-Cre* ([Hebert and McConnell 2000](#_ENREF_4)), early progenitor-active *Emx1-*Cre ([Gorski et al. 2002](#_ENREF_3)), or late progenitor-active *hGFAP-*Cre ([Zhuo et al. 2001](#_ENREF_7)) mouse lines, respectively. Heterozygous animals (i.e., *BAF155^fl^*^/+^, *BAF170*^fl/+^, *Cre*) were used as controls.

***Marmoset Embryos***

*Caesarean Section*

Embryo stage (E)74-pregnant mothers were immobilized by intramuscular injection of 25 µg/head of atropine sulfate (0.5 mg/mL) and 70 mg/kg of ketamine hydrochloride. Thereafter, animals were anesthetized by inhalation of 1–3% of isoflurane via a ventilation mask. Anesthetization management was performed by spontaneous respiration during the operation, monitoring the heart rate and the arterial oxygen saturation. The uterus was exteriorized following midline laparotomy, and the proximal end of the uterus was incised for the Caesarean section. After the Caesarean section, the uterus, abdominal muscles, and skin were sutured. Embryonic brains were dissected in ice-cold phosphate-buffered saline (PBS) and transferred to fixative after removing the meninges.

*Tissue processing*

After dissection, the E74 marmoset brains were immersed in 4% paraformaldehyde (PFA) (w/v) in PBS and left in fixative for 24 h at 4 ^o^C with mild agitation. After fixation, the brains were kept at 4 ^o^C in a mixture of 3 parts PBS containing 0.01% NaN3 and 1 part 4% PFA in 120 mM sodium phosphate buffer with pH 7.4 until processed.

For cryosectioning, fixed brains (marmoset, mouse) were cryoprotected, first in 15% sucrose in PBS and then in 30% sucrose in PBS at 4 ^o^C. Brains were embedded in Tissue-Tek (Sakura Finetek) and stored at -20 ^o^C. Sections were cut at 16 µm and stored at -20 ^o^C.

***Western blot analyses***

Western blot analyses were performed as described previously ([Tuoc and Stoykova 2008](#_ENREF_6)).

***Co-immunoprecipitation (CoIP) and mass spectrometry (MS)***

BAF155 and BAF170 interaction analyses were performed using the neural stem cell line, NS5 ([Conti et al. 2005](#_ENREF_1)), and embryonic telencephalic tissue at E13.5 and E17.5. Tissues were dissected and minced in cold PBS and then washed twice with PBS. Equivalent amounts of cells from one embryo were lysed for 30 min in 1 mLRIPA buffer containing a proteinase inhibitor cocktail (Roche) and DNase. All steps were performed at 4°C. Lysates were centrifuged for 10 min at 13,000 rpm to sediment out non-lysed tissues. The supernatant was pre-cleared by incubating with normal mouse IgG together with protein A/G-agarose beads, as described by the manufacturer (sc-2003; Santa Cruz). Interacting proteins were immunoprecipitated by incubating pre-cleared supernatant with rabbit anti-BAF155 and anti-BAF170 antibodies and A/G-agarose beads. The beads were then washed first with 500 µL cold RIPA buffer (three times for 5 min each) and then with 40 µL of elution buffer (2.5 µL 20% SDS, 5 µL 1 M NaHCO_3_, 42.5 µL double-distilled H_2_O) for 15 min at room temperature.

For MS analyses (performed in the department of Prof. Dr. Henning Urlaub), samples were suspended in NuPage loading buffer and resolved on commercial SDS polyacrylamide gels (Novex NuPage Bis-Tris gel, 4–12% gradient; Invitrogen). Individual lanes were then cut into six squares for MS analysis. The parameters for the identification of proteins were set to the following values: limit, 95% probability of detection; limit of unique peptides detected, 1; and threshold detection probability of peptides, 80%.

The list of BAF155- and BAF170-interacting proteins revealed by MS analysis was obtained by subtracting nonspecific interactions with IgG in IPs and in telencephalic tissues from BAF155-null (*BAF155cKO_FoxG1*-Cre) and BAF170-null (*BAF170cKO_FoxG1*-Cre) mutants. The first set of controls excludes nonspecific binding to the antibody, and the second excludes nonspecific interactions that could possibly be precipitated by either the anti-BAF155 or anti-BAF170 antibody.

**Supplemental references**

Bi-Lin KW, Seshachalam PV, Tuoc T, Stoykova A, Ghosh S, Singh MK. 2021. Critical role of the BAF chromatin remodeling complex during murine neural crest development. PLoS Genet 17: e1009446. doi: 10.1371/journal.pgen.1009446

Conti L, Pollard SM, Gorba T, Reitano E, Toselli M, Biella G et al. 2005. Niche-independent symmetrical self-renewal of a mammalian tissue stem cell. *PLoS Biol* **3**: e283. doi: 10.1371/journal.pbio.0030283

Folgueras AR, Guo X, Pasolli HA, Stokes N, Polak L, Zheng D et al. 2013. Architectural niche organization by LHX2 is linked to hair follicle stem cell function. *Cell Stem Cell* **13**: 314-327. doi:10.1016/j.stem.2013.06.018

Gorski JA, Talley T, Qiu M, Puelles L, Rubenstein JL, Jones KR. 2002. Cortical excitatory neurons and glia, but not GABAergic neurons, are produced in the Emx1-expressing lineage. *J Neurosci* **22**: 6309-6314. doi: 10.1523/JNEUROSCI.22-15-06309.2002

Hebert JM, McConnell SK. 2000. Targeting of cre to the Foxg1 (BF-1) locus mediates loxP recombination in the telencephalon and other developing head structures. *Dev Biol* **222**: 296-306. doi:10.1006/dbio.2000.9732

Kammandel B, Chowdhury K, Stoykova A, Aparicio S, Brenner S, Gruss P. 1999. Distinct cis-essential modules direct the time-space pattern of the Pax6 gene activity. *Dev Biol* **205**: 79-97. doi:10.1006/dbio.1998.9128

Narayanan R, Pham L, Kerimoglu C, Watanabe T, Castro Hernandez R, Sokpor G et al. 2018. Chromatin Remodeling BAF155 Subunit Regulates the Genesis of Basal Progenitors in Developing Cortex. iScience 4: 109-126. doi:10.1016/j.isci.2018.05.014

Tuoc TC, Stoykova A. 2008. Trim11 modulates the function of neurogenic transcription factor Pax6 through ubiquitin-proteosome system. *Genes & development* **22**: 1972-1986. doi:10.1101/gad.471708

Tuoc TC, Boretius S, Sansom SN, Pitulescu ME, Frahm J, Livesey FJ et al. 2013. Chromatin regulation by BAF170 controls cerebral cortical size and thickness. Dev Cell 25: 256-269. doi:10.1016/j.devcel.2013.04.005

Xie Y, Castro-Hernández R, Sokpor G, Pham L, Narayanan R, Rosenbusch J, Staiger JF, Tuoc T. 2019. RBM15 Modulates the Function of Chromatin Remodeling Factor BAF155 Through RNA Methylation in Developing Cortex. Molecular neurobiology 56: 7305-7320. doi: 10.1007/s12035-019-1595-1

Zhuo L, Theis M, Alvarez-Maya I, Brenner M, Willecke K, Messing A. 2001. hGFAP-cre transgenic mice for manipulation of glial and neuronal function in vivo. *Genesis* **31**: 85-94. doi: 10.1002/gene.10008
